# Granulomatous Lymphocytic Interstitial Lung Disease (GLILD) in Common Variable Immunodeficiency (CVID)

**DOI:** 10.5334/jbsr.2944

**Published:** 2022-12-12

**Authors:** Nico Hustings, Adriana Dubbeldam, Birgit Weynand

**Affiliations:** 1 UZ Leuven, BE

**Keywords:** CVID, GLILD, pulmonary, CT, radiology, pathology

## Abstract

**Teaching Point:** Granulomatous lymphocytic interstitial lung disease is a non-infectious complication of common variable immunodeficiency. Computed tomography (CT) has an important diagnostic value by demonstrating pulmonary nodules, ground glass opacities, bronchiectasis, subpleural reticulations, lymphadenopathy and splenomegaly.

## Case History

A 45-year-old woman with known common variable immunodeficiency (CVID) had a history of recurrent upper- and lower respiratory tract infections. She presented to the pneumologist with an acute exacerbation of respiratory symptoms of dyspnea and cough. Velcro-type crackles were observed on clinical examination. Lung function tests revealed a restrictive pattern. Laboratory findings showed stable pancytopenia and decreased titers of IgG, IgM and IgA due to CVID.

**Figure 1:**
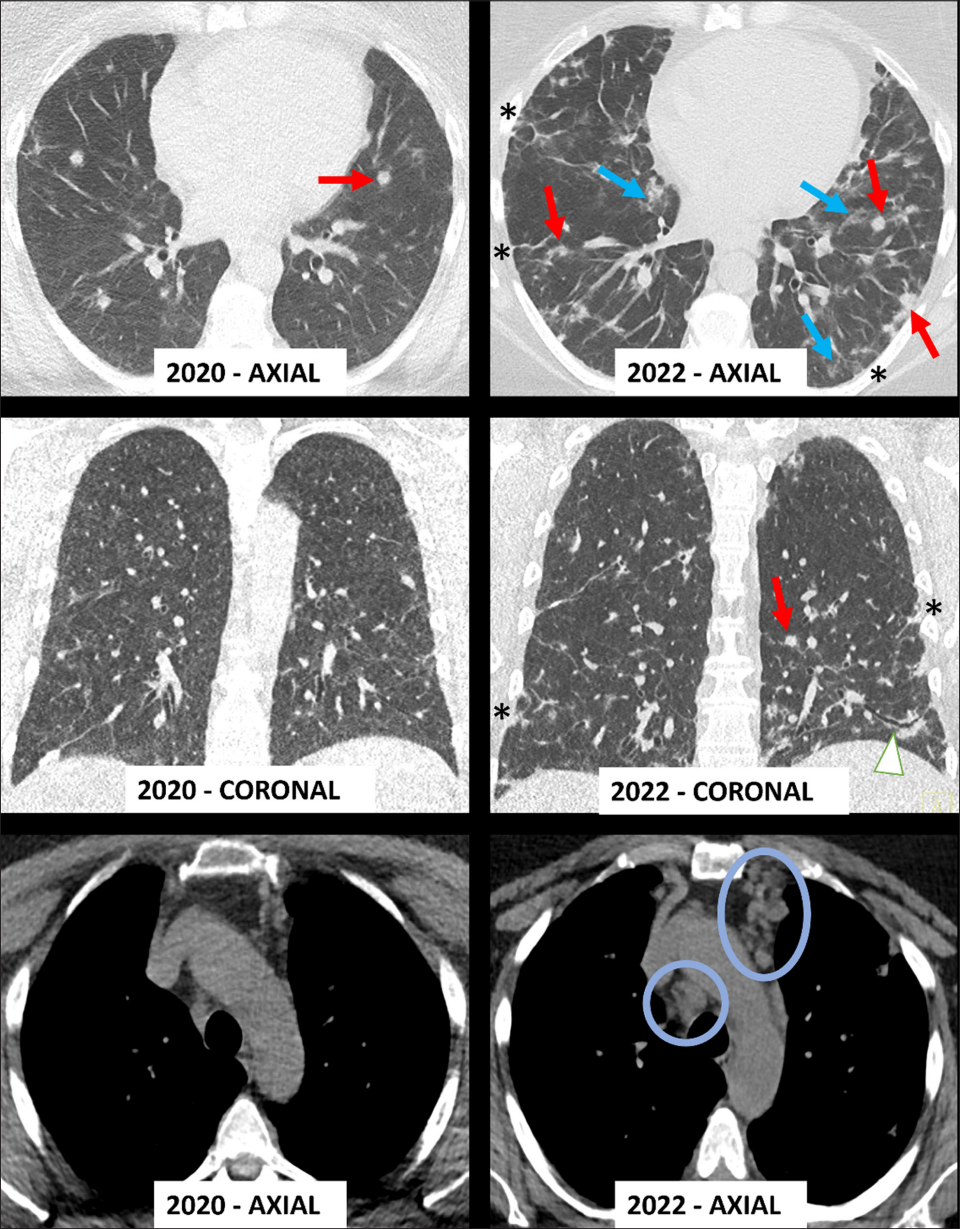


In Figure 1, computed tomography (CT) of the chest revealed progressive lesions compared to previous examination two years earlier. The increased number and volume of multifocal nodules (red arrows) was now accompanied by onset of bronchiectasis (arrowhead), ground glass opacities (blue arrows) and interspersed course reticulations (*). Multiple new mediastinal (blue circles) and hilar (not shown) lymphadenopathy were present.

**Figure 2:**
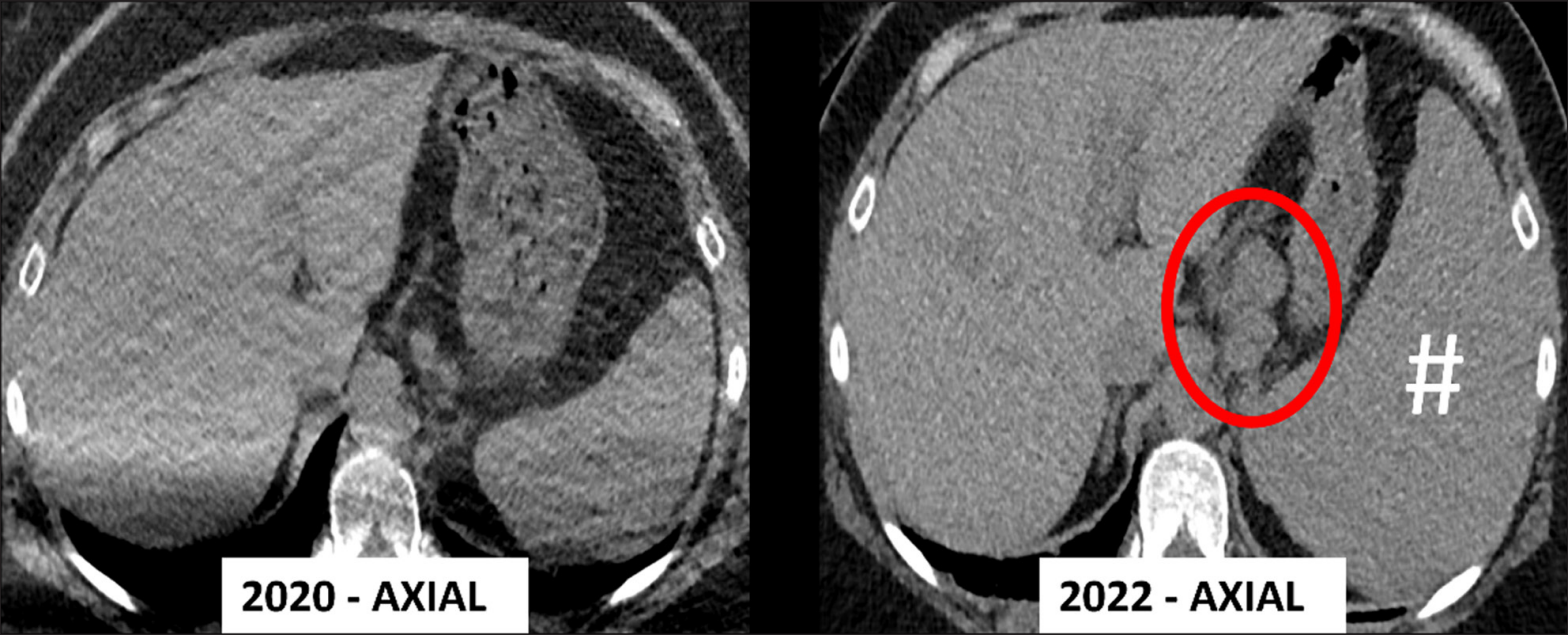


Figure 2 shows additional extrapulmonary findings that were apparent in the visualized part of the upper abdomen, consisting of lymphadenopathy (red circle) and splenomegaly (#).

**Figure 3:**
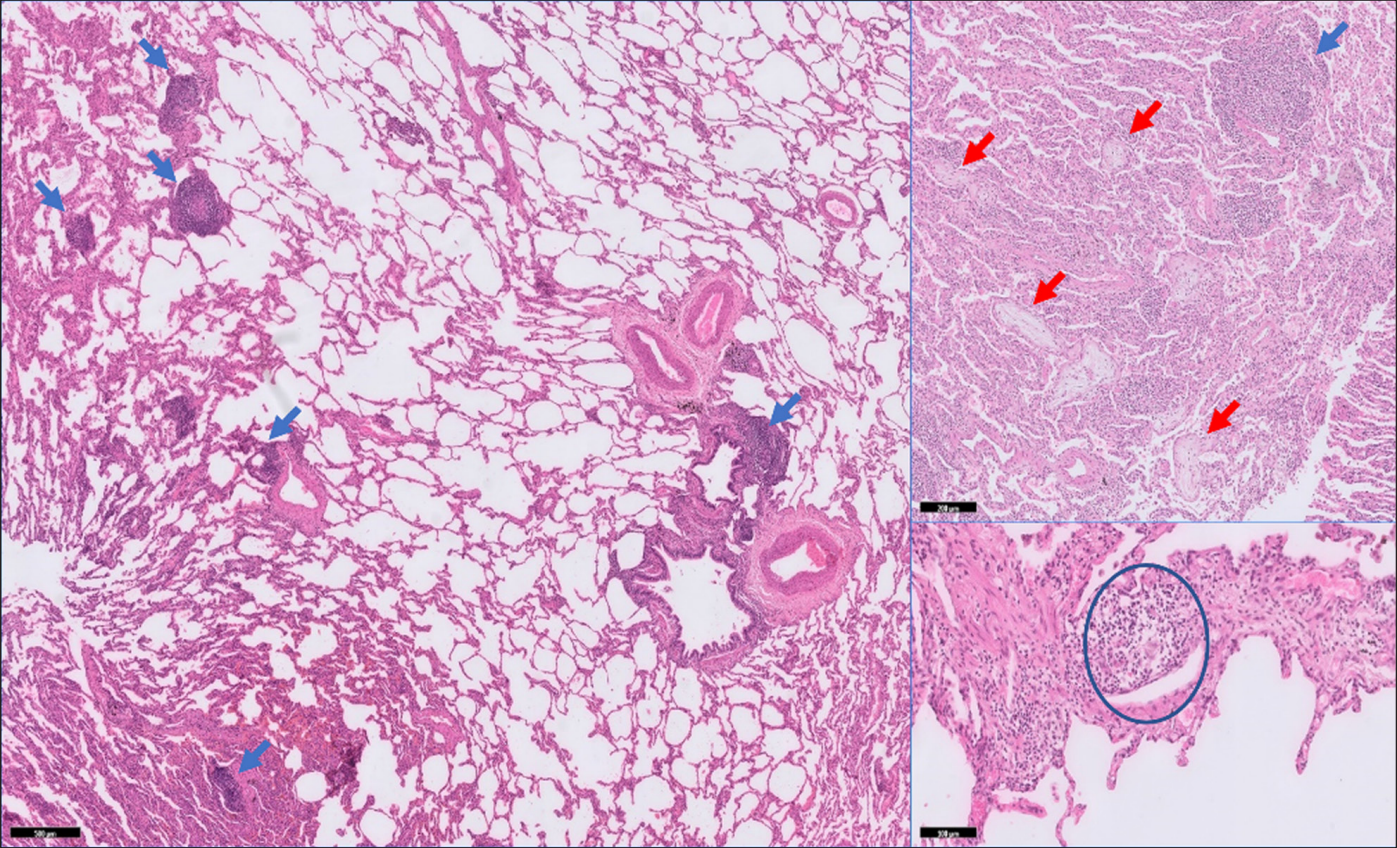


A consecutively performed lung biopsy showed findings consistent with granulomatous lymphocytic interstitial lung disease (GLILD) (Figure 3). Abundant peri-bronchial and peri-bronchiolar chronic inflammation was observed with presence of follicle centers (blue arrows) accompanied by focal intra-alveolar fibroblast plugs consistent with organizing pneumonia (red arrows). Only one microscopic non-necrotizing granuloma could be identified (circle).

## Comment

CVID is a group of immunologic diseases, characterized by lowered IgG, and associated with lowered IgA and/or IgM, and reduced antibody response to infections [1]. Immunoglobulin replacement therapy proved its value by tackling infectious diseases, which has led to a significant decrease of the morbidity and mortality in CVID patients. However, non-infectious complications of CVID are now on the rise. GLILD is a non-infectious lung complication that develops in around 25% of patients with CVID. GLILD has been associated with long-term lung damage and carries an increased risk of lymphoproliferative disorders. Correct diagnosis requires clinical suspicion and radiological and histopathological assessment of the lung. Typical findings on CT include: lung nodules, ground glass opacities, bronchiectasis, reticulations and hilar and/or mediastinal lymphadenopathy. Often the lung findings are associated with extrapulmonary findings such as generalized lymphadenopathies and splenomegaly. Currently there is no consensus on GLILD treatment; depending on the center-expertise, different immunosuppressants (e.g., corticosteroids) are used, though with variable efficacy.
